# Simulation of errors related to the VLBI transmitter on Genesis and their impact on the terrestrial reference frame

**DOI:** 10.1007/s00190-026-02120-2

**Published:** 2026-10-20

**Authors:** Helene Wolf, Lisa Kern, Johannes Böhm

**Affiliations:** Department of Geodesy and Geoinformation, TU Wien, Wiedner Hauptstraße 8-10, 1040 Vienna, Austria

**Keywords:** Very long baseline interferometry, Genesis, Terrestrial reference frame, VLBI transmitter

## Abstract

Mission Genesis of the European Space Agency has been approved for launch in 2028. Its primary goal is the enhancement of the terrestrial reference frame by establishing a space tie on the Genesis satellite between all space geodetic techniques used for the determination of the frame. The satellite will be equipped with a dedicated Very Long Baseline Interferometry (VLBI) transmitter for geodesy. In this work, simulation studies with a global 20-station VLBI network are carried out to assess the impact of errors related to the VLBI transmitter on the terrestrial reference frame, more precisely on station coordinates derived from VLBI observations to Genesis. Our findings indicate that the calibration of the position of the VLBI transmitter on Genesis is critical for the success of the mission. Errors in the position at the one-centimeter-level will cause station coordinate errors of several millimeters. In particular, the tangential and cross-track components do have a significant influence. The precision of the VLBI group delays, however, is not as critical. Simulating white noise for the group delays of 40 ps does still yield station coordinate accuracies at the millimeter level, if estimated in a global solution from weekly 24-h sessions over five years. Considering the investigated aspects, the simulations confirm that the VLBI-related goals of Genesis can be achieved, if stringent requirements with the VLBI transmitter are fulfilled.

## Introduction

The European Space Agency’s (ESA) Genesis mission, scheduled for launch in 2028, is dedicated to the enhancement of the terrestrial reference frame (TRF). It will deploy a satellite integrating several space geodetic techniques (Delva et al. 2023). The satellite will be equipped with Global Navigation and Satellite Systems (GNSS) receivers, a Satellite Laser Ranging (SLR) retroreflector, a Doppler Orbitography and Radiopositioning Integrated by Satellite (DORIS) receiver, and a Very Long Baseline Interferometry (VLBI) transmitter. As of March 2026, the Genesis satellite will operate in a polar orbit at an altitude of about 6000km.

Recent realizations of the International Terrestrial Reference System (ITRS), such as the ITRF2020 (Altamimi et al. 2023) or the DTRF2020 (Seitz et al. 2026), are using local tie measurements at co-location sites for linking the instrument reference points of different techniques within the estimation of global terrestrial reference frames. However, the distribution of these sites is inhomogeneous, and local measurements at the sites are infrequently updated. In addition, technique-specific systematic effects and the challenges in assessing the reference points (e.g., the intersection of axes of large VLBI radio telescopes or phase centers with GNSS antennas) of individual space geodetic techniques using terrestrial measurements are often problematic, making the local tie measurement potentially error-prone (Altamimi et al. 2016; Glaser et al. 2015). For example, over 50% of local tie measurements in ITRF2020 between GNSS and VLBI exhibit discrepancies greater than 5 mm, indicating significant differences between terrestrial measurements and space geodetic estimates (Altamimi et al. 2023). Discrepancies between GNSS and other techniques are even more pronounced, with SLR showing a 64% discrepancy rate and DORIS 68%. Consequently, errors in the local ties on the ground are one of the major limiting factors for the accuracy of terrestrial reference frames.

The Genesis satellite represents a unique, dynamic space geodetic observatory capable of establishing highly accurate and continuous space ties, which refer to relative position vectors between the receiving/transmitting units onboard the satellite. This capability, however, will have to be demonstrated with the mission in orbit. Consequently, all space geodetic techniques (VLBI, GNSS, SLR, DORIS) can be used to estimate the most accurate orbit of Genesis, although it is expected that GNSS is dominant with the large number of continuous observations. Once this orbit is available, VLBI, SLR, and DORIS observations to the Genesis satellite can be used to derive respective and consistent station coordinates in the frame of the Genesis satellite in a second step. This approach will allow for the augmentation and validation of local ties measured on the ground. Furthermore, VLBI observations to the VLBI transmitter on Genesis will enable the direct estimation of the satellite in the celestial reference frame (Böhm and Wolf 2024).

The importance of a high-quality TRF extends beyond achieving the goals of the Global Geodetic Observing System (GGOS), which include a frame accuracy of 1 mm and stability of 0.1 mm/yr (Plag and Pearlman 2009). It is also crucial for determining precise orbits and quantifying the Earth’s rotation, tectonic plate motion, and investigating climate change processes, such as ice mass loss and sea level rise.

In the following, we summarize recent work related to VLBI observations to satellites. While there is experience with GNSS, SLR, and DORIS on satellites, a dedicated VLBI transmitter supporting VGOS observations across four bands is new and thus deserves special considerations. It has to be noted that already in 2015, VLBI observations to the APOD (Atmospheric density detection and Precise Orbit Determination) satellite were carried out (Sun et al. 2018), however, at distinct tones emitted in two bands. Moreover, Tornatore et al. (2014) conducted a single-baseline observation of GLONASS (Global Navigation Satellite System) signals using VLBI telescopes, assessing the feasibility of the technique across the entire operational chain, including scheduling, tracking, acquisition, recording, correlation, and data processing. The technical feasibility of a VLBI transmitter onboard a satellite, including measurement and equipment choices, has been examined by Jaradat et al. (2021) and Karatekin et al. (2023). To test the signal processing feasibility in correlation and fringe-fitting, the VLBI Raw Data Simulator (VieRDS; Gruber 2022) has been utilized to generate the output of a VLBI radio telescope observing a VLBI transmitter. It has been shown that signals from quasars with a flat power spectrum and signals from a transmitter with a distinct power spectrum can achieve similar performance.

The potential of VLBI transmitters on GNSS satellites and Genesis is summarized by Böhm and Wolf (2024). They argue that the geometry of observations to Genesis will be superior to VLBI observations of GNSS satellites, which is due to the lower altitude of Genesis, resulting in a faster moving satellite. This feature would facilitate the estimation of station coordinates and the determination of the orbit. Several studies have focused on determining satellite orbits from VLBI observations. For instance, Kłopotek et al. (2020) demonstrated the feasibility of geodetic VLBI for precise orbit determination (POD) of medium Earth orbit (MEO) satellites and assessed the impact of satellite observations on the quality and quantity of derived geodetic products. Wolf et al. (2022) proposed evaluating satellite observations from VLBI stations in terms of orbit determination using Dilution of Precision (DOP) factors. Furthermore, Wolf et al. (2026a) demonstrated the potential of VLBI observations to satellites for estimating orbital elements and solar radiation pressure parameters, based on partial derivatives computed with the Bernese GNSS Software (Dach et al. 2015). In a recent contribution, Raut et al. (2023) investigated the potential of VLBI transmitters on GNSS satellites for geodetic products. They argue that the network geometry plays a vital role in estimating VLBI station coordinates, satellite orbits, and Earth rotation parameters and that satellite observations added in a classical VLBI session do not cause significant differences in the geodetic results. Furthermore, Wolf et al. (2023) demonstrated the direct estimation of the right ascension of a Galileo satellite in the celestial reference frame and the importance of quasar scans for the determination of the troposphere parameters based on simulated VLBI observations to GNSS satellites. In this context, the possibility of transferring UT1-UTC for satellite orbits was investigated by Sert et al. (2022). Moreover, the concept of co-location satellites in space was examined by Männel (2016) and Herrera Pinzón and Rothacher (2020).

Schunck and Mccallum (2024a) revised the pipeline from planning to analysis of satellite observations (Plank et al. 2017; Hellerschmied et al. 2018) in order to identify the limitations for future satellite experiments and to conduct a visibility study of the Genesis satellite from current and future VGOS networks. In any case, correct modeling of the group delays for near-field targets has to be implemented (Jaron and Nothnagel 2019). Furthermore, various simulation studies have been conducted to explore the concept of frame ties between the kinematic quasar frame and the dynamic satellite frame. In this regard, Plank (2013) and Plank et al. (2014) examined various satellite orbits and antenna networks to determine station coordinates. For the latter, it has been shown that there is a strong correlation between the number of observations to satellites from a VLBI station and the accuracy of weekly station coordinates. In addition, tropospheric disturbances, in particular, have a major influence on the geodetic results if they are not determined accurately. Anderson et al. (2018) examined the establishment of frame ties for a Genesis-like satellite, while Wolf and Böhm (2023) conducted a comprehensive investigation of the optimal distribution of one to three VLBI transmitters in the Galileo space segment for determining frame ties. They recommended three satellites in the same plane and a ratio of satellite observations of 30–40% to ensure an accurate station position estimation along with enough quasar observations for a high sky coverage. Additionally, Schunck and Mccallum (2023) investigated the determination of frame ties based on observations to GPS satellites in the L-band and stated that these experiments were not precise enough to tie together the VLBI and the satellite frame, as station position repeatabilities of about 9 to 13 mm were achieved. They identified the reduced sky coverage as a limiting factor due to the inclination of 55$^\circ$. Recently, Schunck et al. (2024b) assessed the frame tie accuracy between the VLBI and Genesis frames based on station positions estimated solely from satellite observations. In this study, station position repeatabilities of biweekly sessions over 12 months improved to 1.8 mm when orbit errors were not considered. Moreover, Skeens et al. (2024) developed a novel interferometer between a VLBI telescope and a GNSS antenna. This setup has the potential to produce precise local tie vectors directly between the geodetic reference points of the two systems, which are essential for the combination of techniques used to determine terrestrial reference frames. According to Delva et al. (2023), highly precise Genesis measurements could potentially improve the accuracy and stability of the TRF by a factor of 5–10, which would allow the fulfillment of the GGOS requirements. However, several conditions must be met to achieve this goal, including precise calibration of the payloads and the satellite center-of-mass (CoM) and maintaining their offset stability at the <1 mm level, highly accurate orbit determination at the mm level, and a common time reference.

Thus, in this simulation study, we evaluate the impact of transmitter-induced signal noise, bandwidth constraints, and calibration inaccuracies related to the VLBI transmitter on the terrestrial reference frame, i.e., on the station coordinates derived from VLBI observations to Genesis. First, we simulate different levels of white noise of the group delays, which represent different levels of delay precision. However, we will not go into technical details with reasons for certain levels of white noise, and we want to emphasize here that delay precision solely does not guarantee VLBI to Genesis to be successful. There are more technical hurdles, some of which are listed below (see Sect. 2.2.1). Second, we assess the impact of uncertainties in the calibration of the position of the VLBI transmitter on the satellite, i.e., if the position of the phase center of the VLBI transmitter is not perfectly known with respect to the center-of-mass of the satellite. We determine station coordinate repeatabilities in East, North, and Up direction solely from simulated VLBI observations to Genesis in single-session and multi-session solutions in order to assess whether an accuracy of 1 mm can be achieved in the presence of errors related to the VLBI transmitter.

In Sect. 2, a description of the study setup, including scheduling, simulation, and analysis for the investigated cases, is provided. The impact of errors with the VLBI transmitter on Genesis (precision of group delay and calibration errors) on the determination of station positions is presented and discussed in Sect. 3. Finally, we provide a conclusion and outlook in Sect. 4.

## Study setup

### Scheduling

**Fig. 1:**
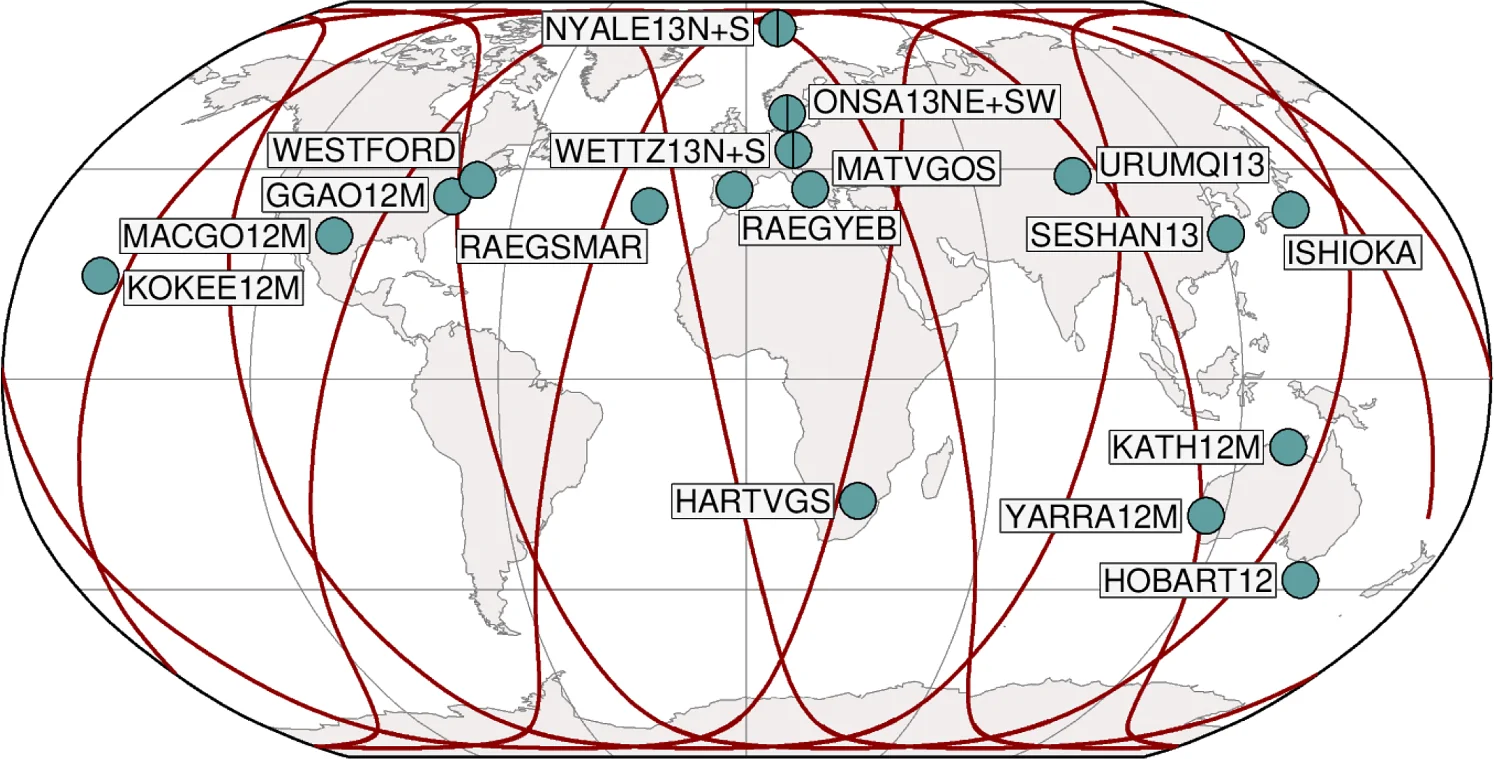
VGOS stations considered in this study and Genesis ground track (red) for 24 h starting on April 4, 2022, at 00:00:00 UTC

This study examines five years of VLBI data from 2020.0 until 2025.0 , with one 24-h session (0:00 UTC to 0:00 UTC) per week (on Mondays), to align with the current VGOS session frequency. A network comprising 20 VGOS stations is used, as illustrated in Fig. 1, to observe radio sources and satellites across four frequency bands ranging from 2 to 14 GHz. While the operational lifespan of the Genesis satellite is planned for two years, this study leverages a five-year dataset for an enhanced analytical depth. Notably, the mission duration is intended to be extendable to up to five years, contingent upon successful operational performance.

The schedules are generated with the scheduling software VieSched++ (Schartner and Böhm 2019) using a 20-station network, comprising three twin stations. No special adjustments were made for these twin stations; consequently, they observe the same sources. VieSched++ incorporates the introduction of the Genesis satellite using a two-line element (TLE) file. The satellite observations are scheduled automatically, and the satellite’s weight is set to a value of 850 to achieve optimal results. This value was chosen based on findings from previous studies (Wolf et al. 2026b) to ensure that satellite scans account for approximately 13–15% of the total number of scans per schedule. This proportion provides a balance between the number of satellite observations required for a reliable estimation of the station coordinates and the preservation of sufficiently homogeneous sky coverage. While too few satellite observations may result in an insufficient number of observations for a reliable determination of the station coordinates, an excessive number of satellite observations may deteriorate the sky coverage of the schedule. This is because satellite observations are inherently constrained by the satellite’s trajectory and can therefore only sample specific directions in the sky at a given time. An excessive number of satellite observations may consequently lead to a less homogeneous sky distribution, which can negatively affect the estimation of the troposphere and, consequently, the determination of the station coordinates. The duration of the satellite and quasar scans is fixed to 30 s, which is a realistic and promising length for VGOS observations (Schartner et al. 2024). It should be noted that variations in scan length could impact the results, as both the signal-to-noise ratio (SNR) and the total number of observations to quasars and satellites are directly affected.

A minimum repeat time, further referred to as MRT, is imposed for observations of the Genesis satellite. Consequently, a subsequent scan to Genesis cannot be scheduled until a certain interval has elapsed, as consecutive observations to Genesis without quasar observations in between lead to a poor distribution of the observations over the station’s local sky, which in turn negatively affects the accuracy of tropospheric estimation. Testing various MRT values identified 1 min as the optimal setting for estimating station coordinates based on satellite observations. The total time allocated for calibration and overhead is set to 5 s, following the simulation study by Schunck and Mccallum (2023). The slewing times correspond to realistic values for the stations. These settings permit the stations to observe many brief scans well distributed across their local skies. Furthermore, the cutoff elevation for quasar and satellite observations is set to 5$^\circ$, and the minimum number of stations for quasar observations is set to three, and for satellite observations, it is set to two stations. In addition, subnetting is allowed, as this strategy is advantageous for the overall sky coverage with global networks. In accordance with the current planning strategy for VGOS sessions, the observation time is not extended despite the availability of idle times.

These scheduling settings result in a mean number of nearly 128200 observations and around 3060 scans per session, both averaged across the 261 schedules. For clarity, the term scan refers to one source being observed by a number of VLBI stations at a time. Around 6.6% of the mean number of observations can be attributed to Genesis observations. The proportion of scans to Genesis is about 14%. However, these percentages are not consistent across all stations. Note that in the following, we focus on the number of scans rather than the number of observations.

#### Observation statistics

**Fig. 2:**
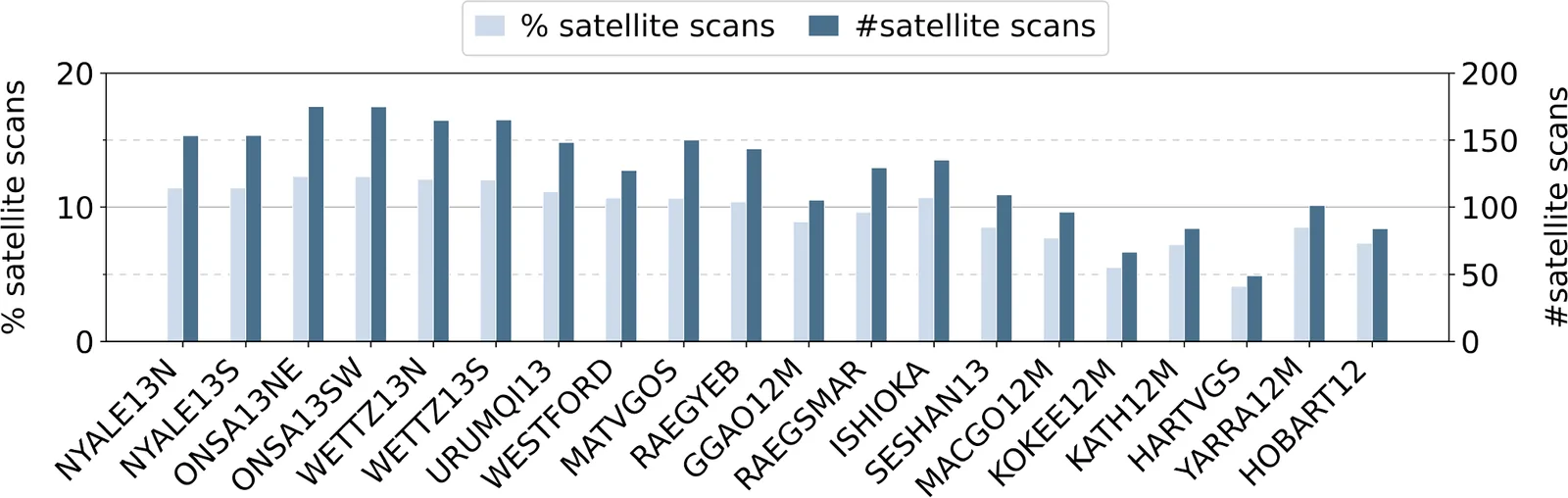
Average number of satellite scans per session (dark blue) and the average ratio of satellite scans of the total number of scans (light blue) over all 261 schedules for the individual stations

Figure 2 shows the average number of satellite scans per session as a total number but also as an average ratio of the total number of scans for the individual stations across all 261 schedules. It can be seen that the VLBI stations in the southern hemisphere, KATH12M, HARTVGS, YARRA12M, and HOBART12, as well as the station KOKEE12M in Hawaii have significantly fewer satellite scans (between 4.0 and 8.5%). In contrast, twin-station pairs such as ONSA13NE/ONSA13SW (Onsala) and WETTZ13N /WETTZ13S (Wettzell) each reach about 12%. For the Australian stations, this reduction can be attributed to the slower slew rates of 5$^\circ$/s in azimuth and 1.25$^\circ$/s in elevation (compared to 12$^\circ$/s and 6$^\circ$/s for WETTZ13N and WETTZ13S, see Table 1), in conjunction with the configuration of the network. Twin stations benefit from being situated in such close proximity. They effectively view the same sky sector, allowing them to observe the same sources simultaneously, which results in a higher number of scans. Conversely, stations in the southern hemisphere lack a considerable number of partners, which restricts their ability to observe the satellite from diverse angles with minimal slewing. A similar geometric constraint affects KOKEE12M, where all baselines to neighboring stations exceed 5 400 km, which results in a lower number of scans. Among Southern Hemisphere sites, YARRA12M records the highest number of satellite scans. This is because the baseline between YARRA12M and HARTVGS is significantly shorter than the baselines connecting HARTVGS to HOBART and KATH12M. Consequently, this relative proximity enables YARRA12M to perform joint observations with HARTVGS more frequently than the other stations. However, the GGAO12M and WESTFORD stations also exhibit slower slew rates in comparison to the remaining stations. Nevertheless, due to their location within the network, they are still capable of achieving an adequate number of satellite scans, with average ratios of 8.89% for GGAO12M and 10.66% for WESTFORD out of the total number of scans. Conversely, MACGO12M and GGAO12M exhibit fewer satellite scans despite their location in the Northern Hemisphere. This reduction is primarily attributed to their greater distance from European stations compared to their neighbor, WESTFORD. However, the orbital geometry imposes a general limitation. Due to the orbital path, the satellite is visible for a shorter period at stations closer to the equator than at those located at higher latitudes. This difference in visibility duration results in fewer total scans for stations close to the equator such as the Australian and South African stations.

**Table 1:** Azimuth and elevation slew rates of VGOS stations

Slew rates ($^{\circ }$/s)		VLBI stations
Azimuth	Elevation	
12	6	HARTVGS, ISHIOKA, KOKEE12M, MACGO12M, MATVGOS, NYALE13N+S,
		ONSA13NE+SW, RAEGYEB, SESHAN13, URUMQI13, WETTZ13N+S
12	4	RAEGSMAR
5	1.16	GGAO12M
	1.25	HOBART12, KATH12M, YARRA12M
3.33	2	WESTFORD

**Fig. 3:**
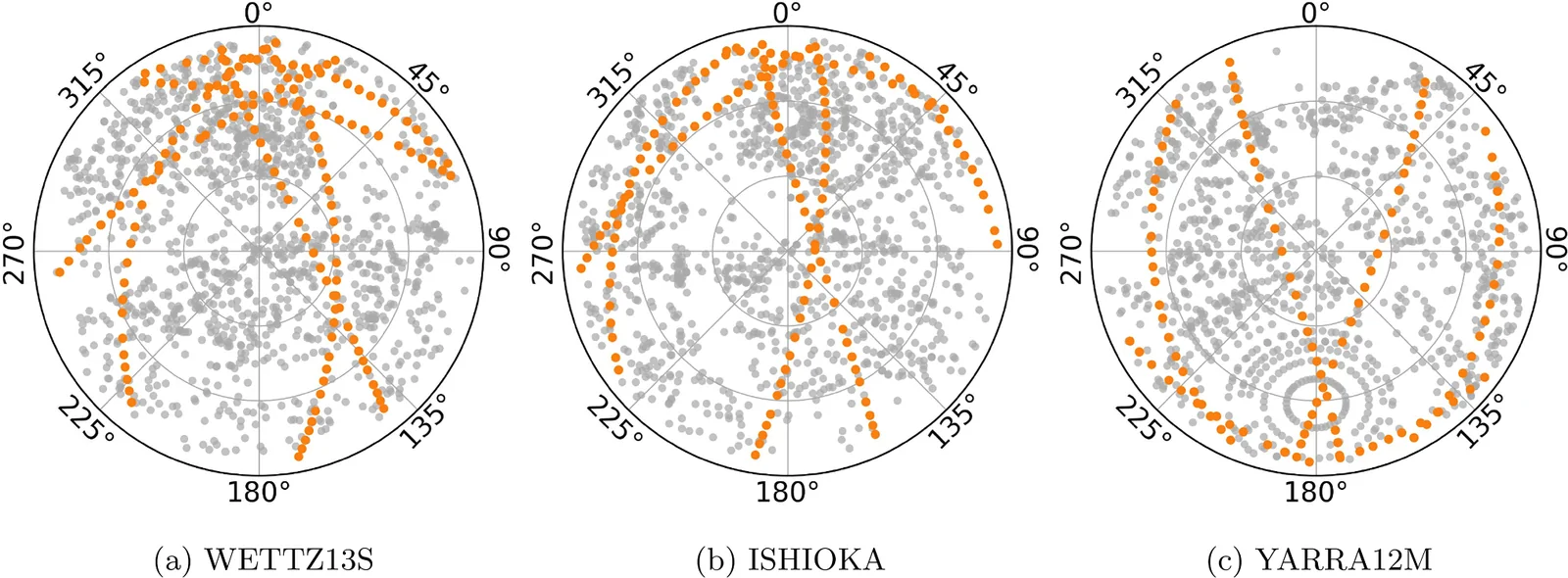
Distribution of the satellite (orange) and quasar observations (gray) on local skies for three stations based on the 24-h schedule on January 6, 2020

To further illustrate the differences in the distribution of the Genesis observations among individual stations, Fig. 3 displays the satellite and quasar observations on the local sky of the stations WETTZ13S, ISHIOKA, and YARRA12M. It is evident that WETTZ13S has a greater number of observations, including more satellite observations, compared to the other two stations. This station is capable of observing the satellite during numerous satellite passes, including those at lower elevations when the satellite is situated at a greater distance. This capability is largely due to the presence of multiple nearby stations that can engage in mutual observations. Additionally, scheduling with its twin station ensures simultaneous observation of the same sources. In contrast, ISHIOKA has less satellite observations than WETTZ13S, primarily due to its proximity to the equator. As noted earlier, the satellite’s orbital trajectory limits visibility duration at lower latitudes, since antennas cannot maintain continuous line-of-sight during polar passes. Consequently, the observation window of ISHIOKA is inherently shorter than that of WETTZ13S. This limitation is even more pronounced in the case of YARRA12M , which is situated closer to the equator than ISHIOKA and lacks nearby stations. Additionally, due to the significantly slower slew rates of this station, the distance between two satellite observations on one pass is larger, as the station requires more time to slew from the satellite to a quasar and then back again.

### Simulation setup

This work comprises two distinct simulation studies. First, we introduce varying levels of white noise into the group delays to model different degrees of delay measurement precision. Second, we evaluate the impact of calibration uncertainties with regard of the VLBI transmitter, specifically examining scenarios where the position of the phase center of the VLBI transmitter is not perfectly known with respect to the center-of-mass of the satellite. Detailed descriptions of these two simulation setups are provided in Sects. 2.2.1 and 2.2.2, respectively.

**Fig. 4:**
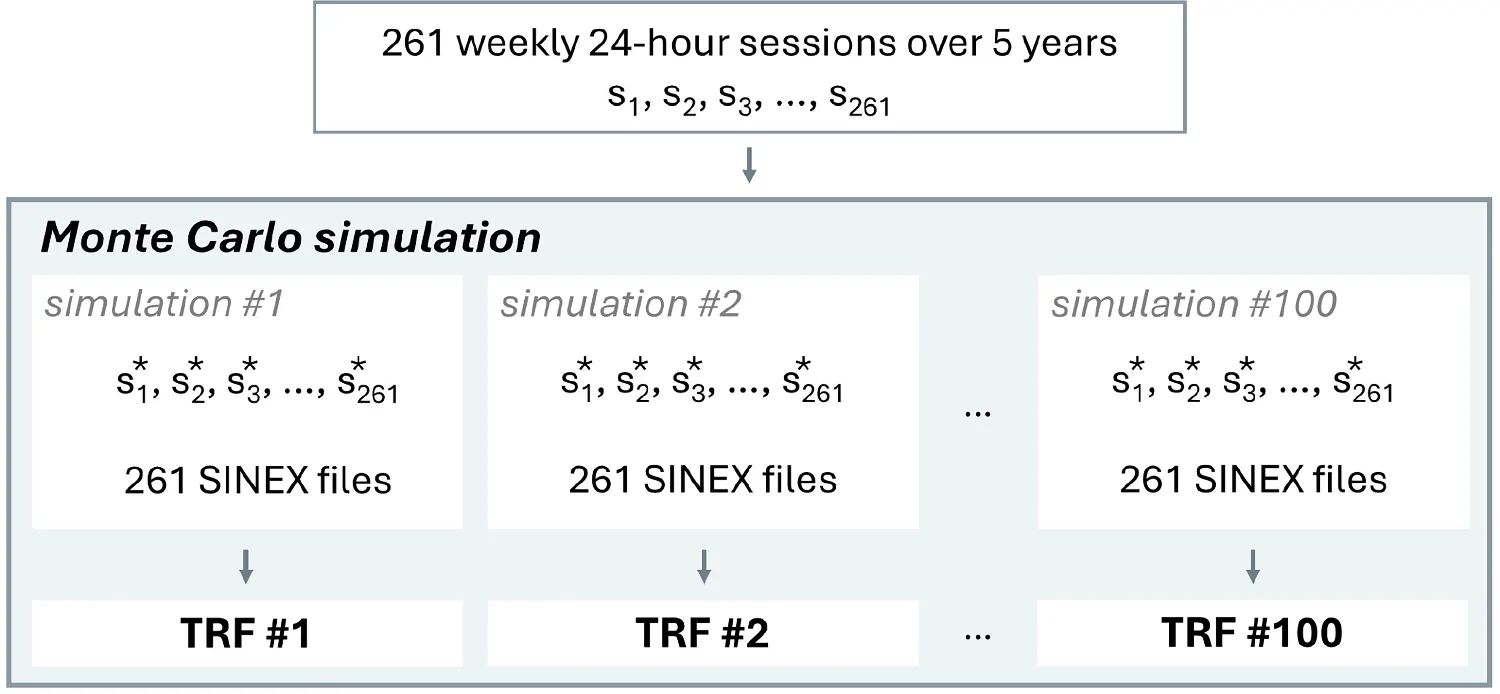
Workflow of the Monte Carlo simulation process. Each of the 261 weekly sessions $(s_1, s_2, s_3,... s_{261})$ is simulated once $(s^*_1, s^*_2, s^*_3,... s^*_{261})$, and a corresponding SINEX file is generated. These 261 simulated sessions are then combined to a TRF realization. This process is repeated 100 times, resulting in 100 TRF realizations, which serve as the basis for a statistical analysis of the repeatability of station coordinates and velocities obtained from the global solutions. The entire procedure is performed for three different white noise levels

#### Precision of group delays for satellite observations

Specifications of the VLBI transmitter aboard Genesis will play a key role for the delay precision to be expected with VLBI observations to Genesis. For example, the frequency setup of the VLBI transmitter on Genesis and the overlap with the distribution of the bands at the ground stations will have an impact. Moreover, the emitted power will also influence the scan lengths, which are necessary to reach certain signal-to-noise ratios (SNR), as the SNR increases with the square-root of the scan length. Scan lengths of 30 s for satellite and quasar observations are consistent with the current VGOS observing strategy.

Other instrumental effects at the transmitter, such as gain curves, and the receiving system as well as imperfections in the signal processing chain including correlation and fringe-fitting will also have an impact on the delay precision (Jaron et al. 2026). For example, the accurate estimation of differential ionospheric delays during the derivation of the broadband delay is challenging (Niell et al. 2018). However, for this investigation, we assume that a VLBI delay measurement can be successfully derived during correlation and fringe-fitting with a specific precision of 10 ps, 40 ps, and 100 ps. This measurement error for quasar and satellite observations is simulated as white noise with different standard deviations, namely 10 ps, 40 ps, and 100 ps. We decided to use the same noise levels consistently for satellite and quasar observations for simplicity. No errors are simulated for the satellite orbit or the space tie on the satellite.

The weekly 24-h schedules are simulated over five years using the three primary error sources: troposphere, clock, and white noise. To investigate the impact of observation errors due to the frequency setup on the ground and in space on the estimated station coordinates, the simulations are carried out for the three different white noise values listed above. The individual schedules are simulated assuming the tropospheric refractive index structure constant C$_{n}$ of 1.8 × $10^{-7}$ m$^{-1/3}$ with a scale height of 2000 m at all stations. The wind speed is simulated with 8m/s in the eastern direction and 0m/s in the northern direction. The clock is simulated as the sum of a random walk and an integrated random walk process, assuming an Allan Standard Deviation of 1 × $10^{-14}$ at 50 min.

The following analysis is conducted for all three white noise values. Each schedule is simulated once and the results from the single-session analysis (VieVS-VLBI) are provided in the form of a single solution independent exchange (SINEX) file per schedule, resulting in 261 SINEX files. These files are utilized in the combination software VieCompy (Kern et al. 2023) to obtain a multi-session or global solution based on the stacking of datum-free normal equation systems. Since the primary goal of the Genesis mission is to improve the TRF, station positions are globally estimated from all sessions by accumulating common values and inverting the normal equation system. As this study comprises a longer investigation period, station velocities are estimated as well, which describe the station movement due to plate tectonics and other geophysical processes. Time-dependent parameters, such as EOPs, clocks, and troposphere, are reduced session-wise from the equations. This process results in two catalogs of coordinates and velocities, with corresponding variance information at a reference epoch and valid within the investigation period—both realized in the terrestrial reference system but one connected to the dynamic reference frame defined by the Genesis satellite orbit, and the other connected to the quasar-based celestial reference frame. In the following, the results for the station coordinates and velocities derived from Genesis observations are investigated in detail.

To mitigate the effect of a single simulation per session, a Monte Carlo simulation is carried out by repeating the aforementioned procedure 100 times, see Fig. 4. This results in 100 independent sets of simulated data covering five years of weekly sessions, from which 100 distinct TRFs are derived. This procedure is performed for three different white noise levels, yielding 100 global solutions per level. The repeatability of station coordinates and velocities is then investigated separately based on the 100 TRFs for each level.

####  Calibration errors

**Fig. 5:**
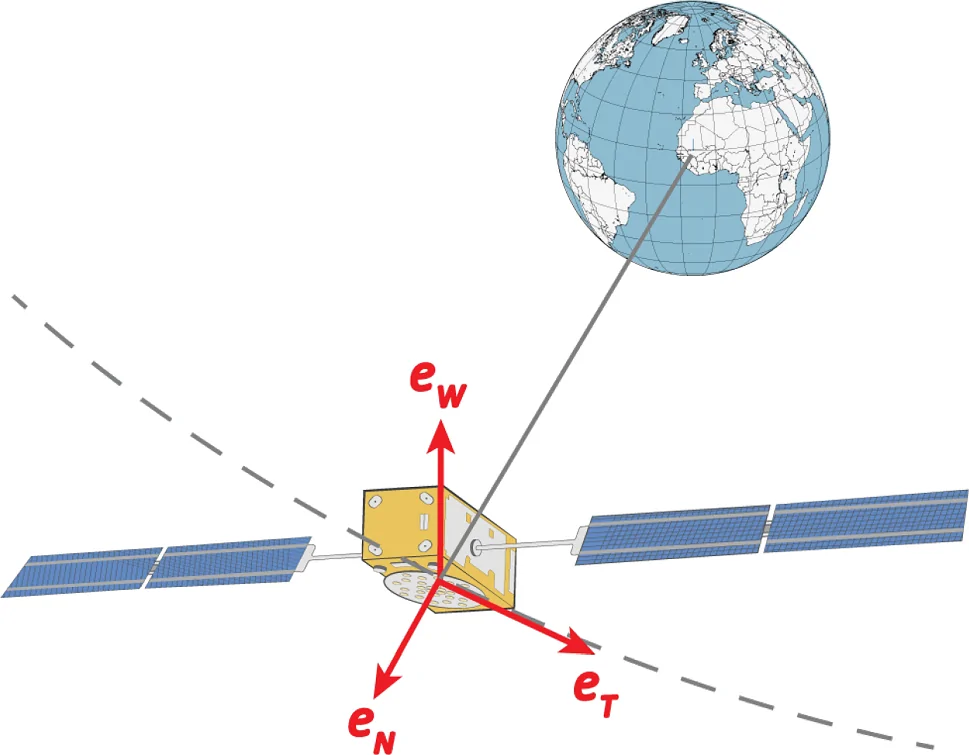
Illustration of the orbit-fixed coordinate system NTW, realized with the unit vectors $\boldsymbol{e_N}$, $\boldsymbol{e_T}$, $\boldsymbol{e_W}$, which are oriented into the normal, tangential, and cross-track directions, respectively

Thus far, we have assumed that the phase center of the VLBI transmitter is in the center-of-mass of the satellite because we do not model the vector between the transmitter and the center-of-mass. In the following, we assume that this vector is not perfectly known and consider shifts of 1 cm in three distinct directions in the orbital frame: normal (N), tangential (T), and cross-track (W) (see Fig. 5). We are aware that these assumptions are simplified. As yaw-steering is anticipated for the satellite, offsets in the satellite-fixed frame will result in varying T and W components. However, with the absence of an attitude model for Genesis, we will keep that assumption in this work.

In order to isolate systematic effects, the simulation excludes the introduction of tropospheric turbulence and clock errors while keeping 10 ps white noise for numerical reasons. Thus, it is not required to stack the results of the five years by obtaining a global solution. Instead, the variability of the station coordinate estimates from the single-session analysis is investigated for the three distinct scenarios involving calibration inaccuracies of 1 cm in the normal, tangential, and cross-track directions. Accordingly, the repeatability is determined based on the 261 individual session estimates.

### Analysis

The schedules are simulated and analyzed with the software package VieVS-VLBI, which is part of the Vienna VLBI and Satellite Software (VieVS; Böhm et al. 2018. In the following, two distinct studies are conducted to investigate the impact of observation and calibration errors on the estimation of station coordinates derived solely from satellite observations. These studies utilize the weekly schedules of 24-h sessions over the five-year period mentioned above, employing varying simulation setups described in detail in Sects. 2.2.1 and 2.2.2. The analysis settings for both studies are identical and based on a least-squares adjustment. The satellite orbit of Genesis is based on a TLE file, and the phase center of the VLBI transmitter is assumed to be in the center-of-mass of the satellite. Moreover, the quasar coordinates and satellite orbit are fixed. The troposphere parameters, including zenith wet delays and northern and eastern gradients, are estimated as piecewise linear offsets (PWLOs) in short intervals using both observation types, namely satellite and quasar observations. The zenith wet delay is estimated in 10-min intervals with relative constraints of 1.5 cm. The northern and eastern gradients are estimated in 20-min intervals with relative constraints of 0.05 cm. The clock at the station with the largest number of observations is designated as the reference clock. The remaining station clocks are estimated based on satellite and quasar observations with a quadratic function and PWLOs in a 60-min interval with relative constraints of 1.3 cm with respect to the reference station. Five Earth Orientation Parameters (EOPs) are estimated from quasar and satellite observations, with daily PWLOs at 0:00 UTC relatively constrained with 1 × $10^{-4}$ microarcseconds. Essentially, for each session, one offset is estimated per parameter.

Finally, the station coordinates and velocities are estimated separately from satellite and quasar observations within a single least-squares adjustment. This results in two sets of station coordinates and velocities realized in the terrestrial reference system—one set is connected to the dynamic reference frame defined by the Genesis satellite orbit, and the other set is connected to the quasar-based celestial reference frame.

The applied datum constraints depend on the estimated parameters and the nature of VLBI measurements. For the set of station coordinates estimated from quasar observations, a no-net-translation (NNT) condition is required, as these observations are insensitive to the network origin. In contrast, satellite observations are sensitive to the origin; therefore, no NNT condition is necessary for the set of station coordinates estimated from satellite observations. However, since Earth orientation parameters (EOPs) are estimated in the analysis, a rank deficiency of three remains. Consequently, no-net-rotation (NNR) conditions are required for both quasar- and satellite-based solutions. In both cases, minimal constraints are applied to avoid network distortions caused by over-constraining. Eventually, the station coordinates are transformed into the topocentric horizon coordinate system with the directions East, North, and Up.

## Results and discussion

### Precision of group delays for satellite observations

**Fig. 6:**
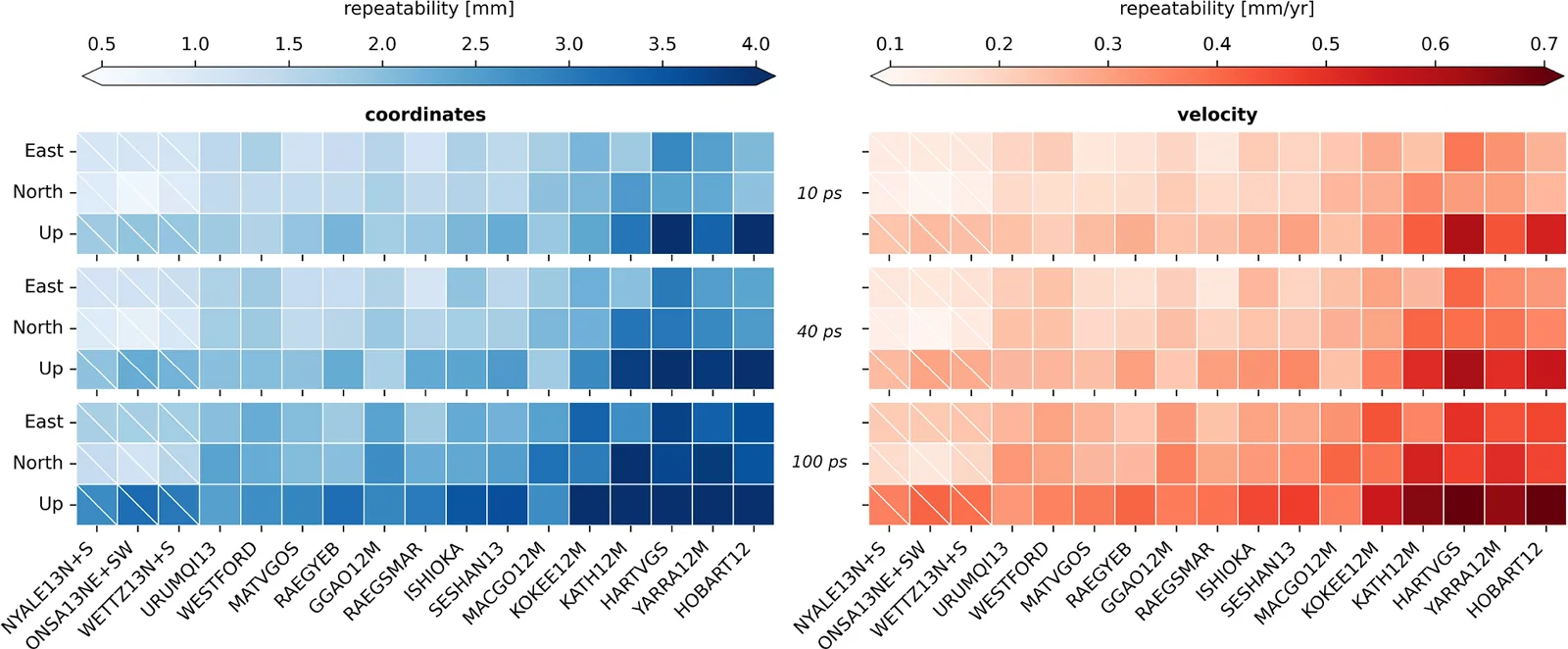
Repeatabilities of the station coordinates (left) and velocities (right) in the directions East, North, and Up derived from a hundred global solutions based on satellite observations with different white noise values of 10 ps, 40 ps, and 100 ps

Figure 6 presents the repeatabilities for the station coordinates and velocities estimated based on satellite observations in East, North, and Up directions of the hundred global solutions for the considered white noise levels. The average repeatability of station coordinates ranges from 0.5 to 2 mm, with a maximum of 6.2 mm. For station velocities, the average repeatability lies between 0.1 and 0.3 mm/yr, with a maximum of 0.83 mm/yr. Overall, the repeatabilities in the North component are smaller compared to the other components. This could be related to a higher sensitivity in this direction, as the satellite’s main direction of movement is North–South. The repeatabilities in the Up component are generally higher, which can be attributed to the strong correlation between tropospheric zenith wet delays and the station height and could indicate that the troposphere is not estimated sufficiently well at some stations (Plank et al. 2014). Notably, the stations on the southern hemisphere KATH12M, HARTVGS, YARRA12M, and HOBART12 exhibit higher repeatabilities, which is likely due to the comparatively lower slew rates, their poorer integration into the station network, and the resulting lower number of satellite and quasar scans at these locations. The solutions based on a white noise value of 40 ps and 100 ps are on average 1.1 and 1.6 times worse in terms of position repeatabilities, respectively, and 1.2 and 1.5 times worse in terms of velocity repeatabilities, compared to the solution based on 10 ps, indicating a nonlinear degradation with increasing white noise values. When comparing the station position repeatabilities from global solutions presented above to those assessed by Schunck et al. (2024b), comparable results are observed. In Schunck et al. (2024b), a repeatability of 1.8 mm was achieved for biweekly VLBI sessions over one year, assuming no orbit errors and a white noise value of 10 ps. However, it is important to note that these studies differ significantly in terms of network, the number of analyzed sessions, and scheduling settings.

As expected, and consistent with the well-known fact that among the three primary error sources simulated in VLBI sessions, the troposphere is the most dominant (Pany et al. 2011), the solution based on 100 ps white noise is worse than that based on 10 ps white noise. However, the overall performance remains primarily limited by tropospheric effects rather than by the white noise level.

The repeatabilities for the station coordinates and velocities estimated based on quasar observations are significantly smaller than those derived from satellite observations. This is expected, as the number of quasar observations per session substantially exceeds that of satellite observations. At white noise levels of 10 and 40 ps, the East and North component repeatabilities range from 0.13 to 0.4 mm, increasing slightly to between 0.2 and 0.7 mm at 100 ps. The Up component repeatabilities are consistently higher across all stations, ranging from 0.5 to 1.2 mm for 10 and 40 ps noise levels, and 0.7 to 1.7 mm for 100 ps. The higher uncertainties in the Up component can again be attributed to the strong correlation between the station height and the tropospheric zenith wet delay.

### Calibration errors

**Fig. 7:**
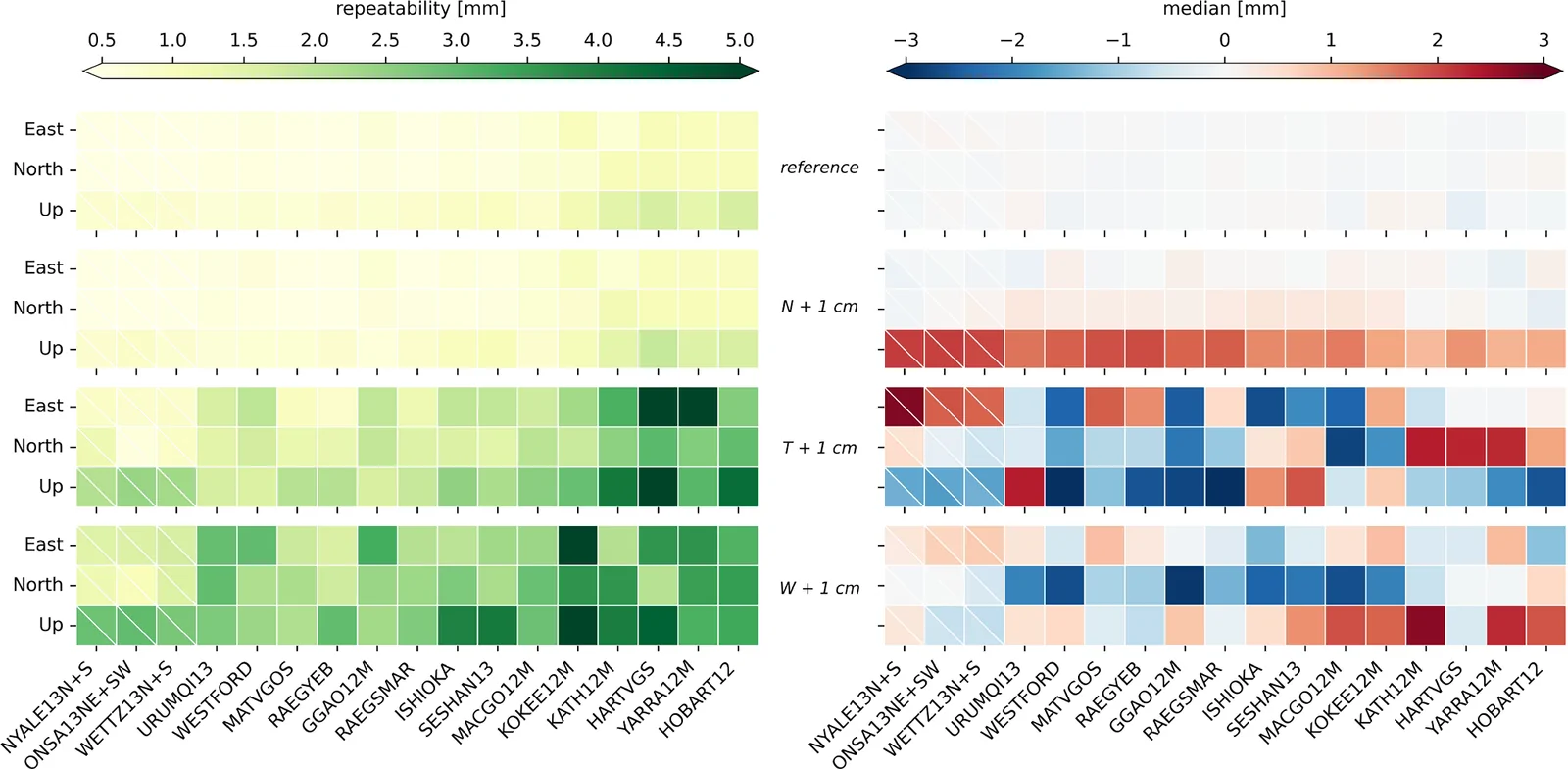
Repeatability (left) and median (right) of the station coordinates in East, North, and Up direction derived from single-session analysis (261 sessions) and a white noise of 10 ps. The first heatmap represents the reference solution with no errors applied. In the second to fourth solutions, constant errors of 1 cm are applied in the N, T, and W directions of the satellite orbit

Figure 7 presents the repeatability and median of the estimates for each coordinate component derived from single-session analysis for a reference solution and each simulated error. The top panel of Fig. 7 shows the results without the introduction of potential calibration inaccuracies, which serves as a reference solution. The repeatability values are between 0.2 and 1.7 mm, which is due to the application of 10 ps white noise, and the median is approximately 0 mm across all components. This test confirms the appropriateness of the simulation setup.

The introduction of a calibration error of 1 cm in N direction (second row of Fig. 7) results in a systematic offset of the Up direction represented by a median offset of 1–2 mm with minor changes in the station coordinate estimates in East and North direction. It is evident that this scenario hardly adds scatter to the solution, as the associated group delay changes are rather small, which is reflected in the low repeatability values that are comparable to those of the reference solution. On the other hand, it is clear that such a calibration error is critical because it can significantly and systematically change station heights by up to 2 mm, causing a scale error of the same size.

In the case of a calibration error of 1 cm in the tangential (T) direction (third row of Fig. 7), distinct features appear. In this scenario, the median values are generally high for most stations and all three components. For example, significant positive offsets of 1.4–2.8 mm in the East component are observed for the twin telescopes in Ny-Ålesund, Onsala, and Wettzell, as well as for MATVGOS. In contrast, pronounced negative offsets ranging from −2.0 to −2.6 mm occur for WESTFORD, GGAO12M, ISHIOKA, SESHAN13, and MACGO12M. Similarly, the North and Up components also exhibit elevated median values across several stations. Overall, the scatter is greater than in the reference solution or in the scenario with a normal-direction error, as the calibration error impacts baselines and epochs differently, with stations in the southern hemisphere exhibiting the largest scatter.

In the case of a calibration error in cross-track (W) direction (bottom row of Fig. 7), systematic negative offsets up to −2.9 mm appear in the North component for European stations, while positive offsets of up to 2.7 mm are observed in the station heights. The scatter is largest among the three calibration error scenarios and is particularly pronounced for stations in the southern hemisphere. For the station KOKEE12M, the repeatability values are the highest, reaching 5.6 mm and 5.3 mm for the East and Up components, respectively. These simulation results clearly confirm the importance of a very accurate calibration of the VLBI transmitter at the mm-level. This level of accuracy is a prerequisite for a terrestrial reference frame as envisaged.

## Conclusions and outlook

In general, several technical hurdles must be overcome for VLBI observations to Genesis to be successful. In this case, it remains to be seen whether the frequency setup, delay precision and accuracy, and calibration of the VLBI transmitter ultimately meet the necessary requirements for VLBI observations to Genesis to enhance geodetic results, such as the terrestrial reference frame.

From a VLBI perspective, it will be important to derive very accurate station coordinates at the 1 mm level or better of radio telescopes from observations to the VLBI transmitter on Genesis. Consequently, requirements for the VLBI transmitter have to be defined. In our simulations, we find that the position of the VLBI transmitter on the satellite has to be calibrated with utmost accuracy. Simulating an error of 1 cm in the tangential and cross-track components of the transmitter’s position yields errors in daily station coordinates of up to a level of half a centimeter. An error in the radial component yields significant and highly systematic errors in the station height component of up to 2 mm. Thus, it is imperative to calibrate the position of the VLBI transmitter with an accuracy of 1 mm. We did not simulate the effect with a yaw-steering satellite as anticipated for Genesis. However, we do not expect different conclusions with that setup. Nor did we simulate orbit errors in this study. The effect, however, is expected to be of similar magnitude. In any case, simulation studies for a yaw-steering satellite and orbit errors have to be carried out in the future.

The available frequencies of Genesis, along with their compatibility with current VGOS modes and station capabilities, will influence the achievable precision of the broadband delay. Testing different levels of white noise, we find that with 40 ps, we can still derive station coordinates in a global solution with 261 sessions with mm-accuracy. However, we certainly want to advocate frequency setups delivering group delays with utmost accuracy.

Note that, in this study, two independent sets of station coordinates are estimated: one based on satellite observations and the other derived from quasar observations. In addition, the impact of constraining the quasar-based station coordinates was explicitly tested, where fixing the station coordinates based on quasar observations does not lead to any significant changes in the station coordinates estimated from satellite observations.

Finally, we want to stress here the limitations of this simulation study. We are using one global station network with a single scheduling strategy, aiming at 13% scans to Genesis. In future, different options have to be tested , particularly regarding the utilization of twin stations. While three twin-station pairs are included in this study, they observe the same sources simultaneously. A more efficient approach could involve subnetting, where one station observes Genesis while its twin observes a quasar. This strategy could significantly increase the observation frequency of the Genesis satellite. Furthermore, also different scenarios with, e.g., two sessions per week, have to be examined. In the future, it is yet to be determined whether Genesis will be observed in dedicated Genesis sessions or be integrated into existing operational VGOS sessions. Furthermore, the frequency of these sessions is contingent upon IVS resource availability, particularly regarding correlation capacity. Finally, the potential impact of satellite observations on the quality of geodetic products remains unexamined. This study focuses exclusively on optimally integrating Genesis observations in a VGOS session, while the geodetic implications remain the subject of future research.

Although the Genesis mission’s operational lifetime is planned for two years, this study examines a five-year dataset. Should the mission fail to extend to five years, the resulting TRF derived from satellite observations would likely exhibit reduced accuracy, reliability, and stability. Furthermore, since velocity estimation typically requires a data span exceeding two years, the reliability and stability of velocity estimates based on a shorter period remain uncertain.

Additionally, it has to be noted that the findings of this study are based on the current state of knowledge as of March 2026.

## Data Availability

The schedules generated and analyzed in this study are publicly available at https://doi.org/10.48436/hsbc4-02b62.
